# Mucin Binding Protein of *Lactobacillus casei* Inhibits HT-29 Colorectal Cancer Cell Proliferation

**DOI:** 10.3390/nu15102314

**Published:** 2023-05-15

**Authors:** Xuan Ju, Xi Wu, Yukun Chen, Shanshan Cui, Zixuan Cai, Liang Zhao, Yanling Hao, Feng Zhou, Fang Chen, Zhengquan Yu, Dong Yang

**Affiliations:** 1 Beijing Key Laboratory of Functional Food from Plant Resources, College of Food Science & Nutritional Engineering, China Agricultural University, Beijing 100083, China; s20193060968@cau.edu (X.J.); yukun1109@cau.edu.cn (Y.C.); sy20183060967@cau.edu.cn (Z.C.); zf@cau.edu.cn (F.Z.); 2 State Key Laboratory of Agrobiotechnology, College of Biological Sciences, China Agricultural University, Beijing 100193, China; sz20183020200@cau.edu.cn (X.W.); zyu@cau.edu.cn (Z.Y.); 3 College of Food Science and Nutritional Engineering, National Engineering Research Centre for Fruits and Vegetables Processing, Key Laboratory of Fruits and Vegetables Processing Ministry of Agriculture, Engineering Research Centre for Fruits and Vegetables Processing, Ministry of Education, China Agricultural University, Beijing 100083, China; cuishanshan@ccmu.edu.cn (S.C.); chenfangch@sina.com (F.C.); 4 Beijing Advanced Innovation Center for Food Nutrition and Human Health, Beijing Engineering and Technology Research Center of Food Additives, School of Food and Health, Beijing Technology and Business University, Beijing 100048, China; liangzhao@btbu.edu.cn; 5 Key Laboratory of Precision Nutrition and Food Quality, Department of Nutrition and Health, China Agricultural University, Beijing 100083, China; haoyl@cau.edu.cn

**Keywords:** *L. casei*, mucin binding protein, SCFAs, anti-proliferative, colorectal cancer

## Abstract

Many *Lactobacillus casei* strains are reported to exhibit anti-proliferative effects on colorectal cancer cells; however, the mechanism remains largely unknown. While there has been considerable interest in bacterial small metabolites such as short chain fatty acids, prior reports suggested that larger-sized molecules mediate the anti-proliferative effect of *L. casei*. Here, other possible ways of communication between gut bacteria and its host are investigated. LevH1 is a protein displayed on the surface of *L. casei*, and its mucin binding domain is highly conserved. Based on previous reports that the cell-free supernatant fractions decreased colorectal cell proliferation, we cloned the mucin binding domain of the LevH1 protein, expressed and purified this mucin binding protein (MucBP). It has a molecular weight of 10 kDa, is encoded by a 250 bp gene, and is composed primarily of a β-strand, β-turns, and random coils. The amino acid sequence is conserved while the 36th amino acid residue is arginine in *L. casei* CAUH35 and serine in *L. casei* IAM1045, LOCK919, 12A, and Zhang. MucBP^36R^ exhibited dose-dependent anti-proliferative effects against HT-29 cells while a mutation of 36S abolished this activity. Predicted structures suggest that this mutation slightly altered the protein structure, thus possibly affecting subsequent communication with HT-29 cells. Our study identified a novel mode of communication between gut bacteria and their host.

## 1. Introduction

Gut microbiota plays an increasingly important role in human physiology, and it is generally believed that short chain fatty acids (SCFAs) produced by gut microbiota bridge their function to the host [1,2,3,4,5]. The production of SCFAs in the gut lumen and their hepatic utilization results in a concentration gradient, decreasing from the lumen to the periphery, where selective uptake of butyrate at the epithelium, propionate at the liver, and acetate in the periphery takes place [6,7,8]. It is certainly unlikely that so many physiological outputs are mediated by the limited number of SCFAs alone. However, it is not yet discovered whether there are other patterns of interactions between gut microbiota and the host [9]. Hence, finding other ways of biological intervention between the gut microbiota and its host not only helps us understand the symbiosis at the molecular level but also fully explore their prebiotic effects.

The outer colonic mucus layer is where the symbiotic bacteria reside, with large gel-forming mucin being the major component [10]. *L. casei* is one of the earliest residents of the human colon at the surface of the mucin layer [11]. As one of the lactic acid bacteria, it exhibits multiple probiotic effects: maintaining the intestine integrity, preventing intestinal tumorigenesis, inducing apoptosis in gastric cancer cells, reducing hepatic inflammation, regulating immune responses, lowering the blood lipids and cholesterols, alleviating insulin resistance and diabetes, and ameliorating learning and memory impairments [12,13,14,15,16,17,18,19,20]. Accumulating evidence shows the anti-proliferative effect of *L. casei* strains against colorectal cancer cells; however, detailed mechanisms of how the bacteria communicates with its host remain poorly understood. There is usually a protein expressed on the outer surface of the bacteria that binds to mucin in the colon, as found in *Streptococcus pneumoniae*, *Lactobacillus acidophilus*, and *Lactobacillus fermentum* [21,22,23,24]. In *Lactococcus lactis* subsp., two types of adhesion proteins are involved in the colonization of bacteria in the gastrointestinal tract [25]. The AggL protein contributes to the bacterial non-specific hydrophobic adhesion to mucous tissues, while a mucin binding protein (MucBP) binds to gastric-type mucin proteins [25]. In all the *L. casei* strains with a known genome, there is an *LevH1* gene coding protein with domains of GH32 inulinase activity, mucin binding activity, and an Ig-like domain. It is highly likely that the mucin binding activity domain in the LevH1 protein on the surface of *L. casei* binds with mucin of the hosts. 

The administration of *L. casei* prevented atypia of colorectal tumors in humans [26]. In the inflamed mucosa, *L. casei* could reduce the number of CD4 cells and TNF-α expression among intraepithelial lymphocytes but did not induce any change in non-inflamed mucosa [27]. Meanwhile, both the supernatant and the extract of *L. casei* decreased Caco-2 cell proliferation and increased cell apoptosis, while its extract also led to cell necrosis [28]. Additionally, both live and irradiation-inactivated *L. casei* increased the apoptosis efficacy of a colorectal cancer cell LS513 induced with 5-fluorouracil (5-FU); however, microwave-inactivated *L. casei* reduced its apoptosis capacity [29]. Heat-killed *L. Casei* MG4584 inhibited the human colorectal carcinoma RKO cells in vitro and in xenograft models [30]. Further, cell-free *L. casei* 21L10 attenuated LPS-induced inflammation and might exert anti-proliferative effects in HT-29 cells [31]. All these evidences indicated that there were some structural components of the bacteria that exhibits antiproliferative activity against colorectal cancer cells, regardless of the live state of the bacterium. Later, it was reported that the cell-free supernatant of *L. casei* decreased colorectal cell invasion primarily via the >100 kDa and 50–100 kDa fractions, suggesting the inhibitory compound(s) might be macromolecules such as proteins, nucleic acid, or a polysaccharide [32]. As proteins usually undergo denaturation via microwave treatment, it is highly possible that the structural component exhibiting antiproliferative activity is a protein. Currently, all the studies are suggesting other mechanisms of antiproliferative effects exerted by *L. casei*, but none of them has pointed out which bacterial component plays the role. Here, the physiological effects of MucBP on colorectal cancer cells were studied to evaluate a possible new communication mode between *L. casei* and its host.

## 2. Materials and Methods

### 2.1. Molecular Cloning, Site-Directed Mutagenesis, and Protein Purification

The *LevH1* gene sequences (GeneBank, AB185852.1) from different *L. casei* strains were searched and annotated to different functions, and the region of the *MucBP* sequence was used to design cloning primers: 5′CATCATCATCATCATTCTTCTGGTATTACTTCAATTTGGAACAGTACTG3′ and 5′CTCCTGGATCTCGCGCTCATACAACAGACGCTTGGGACG3′. *L. casei* CAUH35 was used as the template to amplify the *MucBP* gene with an annealing temperature of 56.7 °C in the presence of Pfu DNA polymerase (Sangon Biotech, Shanghai, China). The amplified *MucBP* gene was purified with agarose gel electrophoresis, and integrated to the pET-30b plasmid with an annealing temperature of 60.8 °C. Primers 5′TAGGACAAAGCTACACTTCTG3′ and 5′CAGAAGTGTAGCTTTGTCCTA3′ were used for site-directed mutagenesis on a PCR instrument with an annealing temperature of 57 °C [33].The plasmid was sequenced, and the verified plasmid was transformed into *E. coli* BL21 (DE3) cells for protein expression under the induction of isopropyl-β-d-thiogalactopyranoside (IPTG, Shanghai Macklin Biochemical Co. Ltd., Shanghai, China). The over-expressed MucBP protein was purified essentially in the same way as described previously [34].

### 2.2. Protein Sequence Alignment

The consensus regions of the *MucBP* gene in *L. casei* IAM1045, *L. casei* LOCK919, *L. casei* 12A, *L. casei* Zhang, *L. casei* ATCC334, and *L. paracasei* CAUH35 were aligned and analyzed as described previously [35].

### 2.3. Protein Structure Characterization

The purified 0.3 mg/mL MucBP protein was dialyzed into 10 mM phosphate buffer saline (PBS) buffer, and its circular dichroism (CD) spectrum was recorded on a Chirascan circular dichroism polarimeter (Applied Photophysics, Leatherhead, UK) by scanning the sample from 200 to 260 nm at 25 °C with a bandwidth of 1 nm and step size of 1 nm, as described previously [36]. Proportions of the secondary structures of the protein were calculated with the Chirascan software (Applied Photophysics, UK). The I-TASSER and RoseTTAFold servers were used to predict the three-dimensional structure of MucBP 36S and 36R variants, as described previously [37,38]. The predicted structures were validated with the ProSA-web Protein Structure Analysis server and the SAVES v6.0 server [39,40,41,42].

### 2.4. Cell Culture

The human colon adenocarcinoma cell line HT-29 used in this study was obtained from the National Infrastructure of Cell Line Resource and cultured at 37 °C in a humidified 5% CO_2_ incubator using RPMI-1640 medium (Gibco, New York, NY, USA, 11875093) supplemented with 10% fetal bovine serum (FBS, Gibco, 16140089) and 1% penicillin–streptomycin (Sigma, St. Louis, MO, USA, V900929).

### 2.5. Cell Viability Assay

The effect of different concentrations (from 5 μg/mL to 200 μg/mL) of MucBP^36R^ and MucBP^36S^ on HT-29 cells viability was assessed using the Enhanced Cell Counting Kit-8 (CCK8; Beyotime Biotechnology, Shanghai, China, C0042) according to the manufacturers’ protocol. HT29 cells were treated with MucBP^36R^ and MucBP^36S^ at different concentrations (5, 25, 50, 100, and 200 μg/mL) for 72 h and then seeded onto 96-well plates (approximately 3 × 10^3^ cells per well containing 100 μL of culture medium) overnight. The supernatant was removed, and 10 μL CCK8 solution with 100 μL fresh medium was added to each well before the plate was incubated at 37 °C for 2 h. The absorbance of each well was measured at 450 nm on a microplate reader (TECAN, infinite F200). The Dunnett’s multiple comparisons test was used to compare experimental groups with the control group.

## 3. Results

### 3.1. Cloning, Purification, and Sequence Comparison of MucBP from L. casei

**Figure 1:**
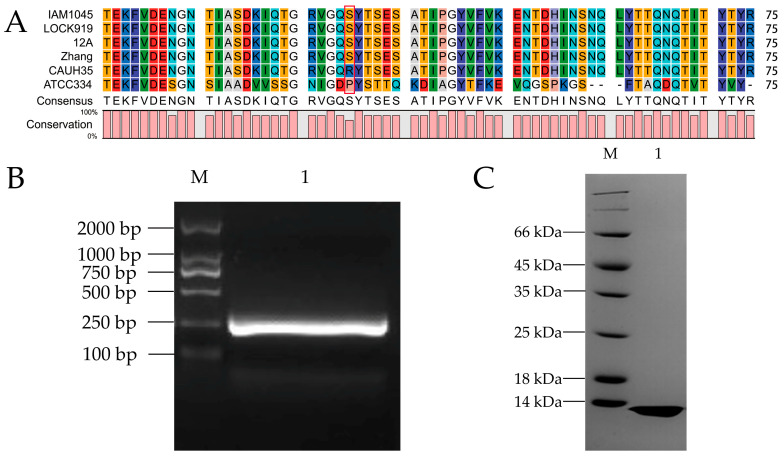
Sequence alignment, cloning, and purification of MucBP from *L. casei*. (**A**) Sequence alignment of the core regions of all reported *MucBP* genes from *L. casei* IAM1045, *L. casei* LOCK919, *L. casei* 12A, *L. casei* Zhang, *L. casei* CAUH35, and *L. casei* ATCC334. Red indicates amino acid residues D, E; yellow indicates C, M; blue indicates R, K; violet indicates F, Y; dark green indicates I, L, V; cyan indicates Q, N; light grey indicates A; tawny indicates P; orange indicates S, T; mauve indicates W; white indicates G; pale blue indicates H. Red square indicates the conserved serine/arginine residue. (**B**) Cloning of the *MucBP* gene shown on an agarose gel. M is the DNA molecular marker, and lane 1 is the cloned *MucBP* of 246 bp. (**C**) Purification of the MucBP protein shown on an SDS-PAGE gel. M is the protein molecular marker, and lane 1 is the purified MucBP protein of 10.3 kDa.

The gene sequence of *LevH1* in *L. casei* CAUH35 was identified (GeneBank, AB185852.1), and the amino acid sequence was analyzed with BLAST, which was highly conserved as in *L. casei* IAM1045 [43]. There are 1294 amino acid residues in the LevH1 protein, and the MucBP domain ranges from amino acid residue 745 to 808. There is a KxYKxGKxW signal peptide in the N-terminal of the LevH1 protein and an LPXTG cell wall anchoring domain in the C-terminal, suggesting that this protein may be secreted and anchored to the *L. casei* cell wall. Following the signal peptide is the inulinase domain, which is supposed to hydrolyze the prebiotic inulin, and then the predicted mucin binding domain. Compared with all the *L. casei* strains with reported genomes, including *L. casei* IAM1045, *L. casei* LOCK919, *L. casei* 12A, *L. casei* Zhang, *L. casei* CAUH35, and *L. casei* ATCC334, the core region of MucBP is highly conserved (Figure 1A). It is noteworthy that the 36th amino acid residue is serine in most of these bacteria, while it is arginine in *L. casei* CAUH35 and proline in *L. casei* ATCC334.

Based on the MucBP domain of the LevH1 protein from *L. casei* CAUH35, a pair of primers was designed to amplify the gene. Agarose gel electrophoresis was employed to purify the gene sequence, and a band of around 250 bp was visualized (Figure 1B), which was consistent with the theoretical 246 bp. Site-directed mutagenesis was used to study the effect of mutation on the 36th amnio acid residue, and the mutation was examined with DNA sequencing.

The *MucBP* sequence was inserted into the pET-30b plasmid, transformed into *E. coli* BL21(DE3) cells, induced with IPTG, and MucBP was purified with Ni-NTA agarose chromatography. Finally, the purified protein was examined with SDS-PAGE gel electrophoresis, and a band below 14 kDa was observed, which was consistent with the theoretical molecular weight of MucBP of 10.3 kDa (Figure 1C). The calculated isoelectric point of this protein is 6.63, and molar extinction coefficient is 12,950 M^−1^cm^−1^. There are 10 serine and 13 threonine residues in this protein.

### 3.2. Secondary and Tertiary Structures of MucBP from L. casei

**Figure 2:**
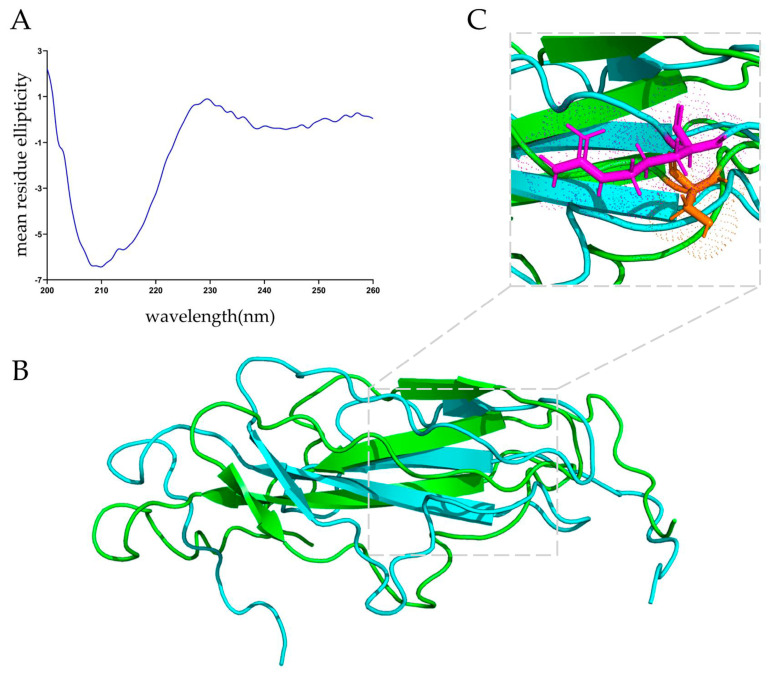
Secondary and tertiary structures of MucBP from *L. casei*. (**A**) Circular dichroism spectrum of the MucBP protein in phosphate buffer. (**B**) Predicted structures of the MucBP proteins. Blue indicates the MucBP^36R^ and green indicates the MucBP^36S^. (**C**) Overlay of the MucBP^36R^ and MucBP^36S^ at the 36th amino acid residue. Purple stick indicates the arginine residue side chain, and orange stick indicates the serine residue side chain.

The purified MucBP^36R^ protein was subjected to circular dichroism analysis for determining its secondary structure. There was a minimum peak at around 210 nm, indicating a mixture of mainly β-strands and disordered regions (Figure 2A). Quantitative analysis indicated there was 29.4% β-strands, 17.7% β-turns, and 41% random coils within this protein.

The I-TASSER and RoseTTA fold servers were employed to predict the tertiary structure of MucBP^36R^, and a globular protein containing majorly β-strands and random coils was generated (Figure 2B). The predicted structures were almost identical at the folded region, and were validated with ProSA-web and SAVES v6.0, respectively. Based on the validation results, structures predicted with I-TASSER were subjected to the following analysis, and MucBP^36R^ displayed positive, negative, and hydrophobic characters on its surface, roughly consistent with the secondary structure analysis.

The MucBP^36S^ protein structure was also predicted, and compared with the 36R variant (Figure 2B). There was a slight structural deviation induced by this mutation, as an RMSD value of 1.036 Å was obtained between these two proteins. The side chain of the 36th serine is pointing differently than the arginine side chain, whose orientation highly likely induced the structural difference between these two protein variants (Figure 2C).

### 3.3. Antiproliferative Activity of MucBP from L. casei

**Figure 3:**
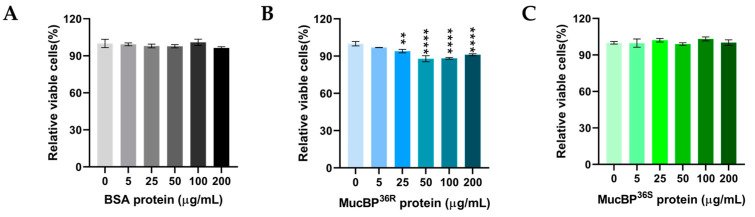
HT-29 cell viability after treatment with various concentration of proteins. Viabilities of HT-29 cells after co-incubation with 0 μg/mL, 5 μg/mL, 25 μg/mL, 100 μg/mL, and 200 μg/mL of BSA (**A**), MucBP^36R^ (**B**), and MucBP^36S^ (**C**), respectively. Darker color indicates higher protein concentration in each panel. The Dunnett’s multiple comparisons test was used. ** indicates 0.01 > *p* value > 0.001, and **** indicates *p* value < 0.0001.

Different proteins were co-incubated with HT-29 cells to evaluate their possible physiological action on this human colorectal cancer cells. The addition of 0, 5, 25, 50, 100, and 200 μg/mL bovine serum album (BSA) protein and co-incubation for 72 h did not show any effect on HT-29 cell proliferation (Figure 3A). The same co-incubation was performed with MucBP^36R^ and MucBP^36S^ proteins. Initially, MucBP^36R^ did not show any effect on HT-29 cell growth at 5 μg/mL. Surprisingly, MucBP^36R^ began to inhibit the proliferation of HT-29 cells at 25 μg/mL, and the inhibition effect was further enhanced at higher protein concentrations (Figure 3B). On the contrary, MucBP^36S^ did not exhibit any effect on HT-29 cell proliferation up to a protein concentration of 200 μg/mL (Figure 3C).

## 4. Discussion

We turned to the surface layer protein MucBP in *L. casei* as a potential candidate exerting anti-proliferative activity against colorectal cancer cells. Although our purified protein was smaller than the predicted >50 kDa functioning fraction, it is only a domain of the LevH1 protein, which was more than 10 folds larger. Thus, it is likely that the MucBP protein is virtually the functioning element in the >100 kDa protein fraction found in the previous study [32]. In bacteria with similar mucin binding capacity, anti-proliferative activity could be found. For example, *L. paracasei* K5 inhibits Caco-2 cell proliferation and displays efficient adherence capacity to this colon cancer cell, similar to the strains *L. casei* ATCC 393 and *L. rhamnosus* GG [44]. Our study is the first one, to the best of our knowledge, proving that the cell surface protein MucBP inhibits human colorectal cancer cell proliferation.

Mechanism wise, it was reported that *L. casei* ATCC 393 exhibited a dose- and time- dependent anti-proliferative effect on HT-29 cells by up-regulating TNF-related apoptosis [45]. *L. casei* BL23 inhibited colorectal cancer growth via the IL-2 signaling pathway [46,47]. For other types of cancer, cell-free culture supernatant of *L. casei* SR1, SR2, and SR4 isolated from human milk inhibited cervical cancer by up-regulating the expression of apoptotic genes [48]. *L. casei* Shirota enhanced the antiproliferative effect of geniposide in human oral squamous carcinoma HSC-3 cells [49]. Here, we show the concentration-dependent antiproliferative effect of MucBP against human colorectal cancer HT-29 cells, which demonstrated the cancer cell inhibition activity of this protein, regardless of whether it is on a cell or in a solution. Compared to the BSA group, protein addition alone is not sufficient to induce the inhibiting effect via perturbation of cell culture conditions. This is in line with observations that surface layer proteins from *L. casei* Zhang, *L. rhamnosus*, *L. gasseri,* and *L. acidophilus* NCFM protected the HT-29 cells from H_2_O_2_-induced oxidative injury [50]. It is obvious that surface layer proteins could interact directly with HT-29 cells. Although we did not investigate the detailed mechanism of how MucBP inhibits HT-29 cell proliferation, the phenomenon that a single mutation of arginine into serine at the 36th amino acid undermined the anti-proliferative capacity of MucBP suggested that there might be a receptor on the surface of HT-29 cells that could bind with MucBP and induce colorectal cancer cell death.

Our study does not intend to limit the probiotic effect of *L. casei* only to the MucBP-inhibited colorectal cancer cell proliferation. There are also other bacteria-related, small or big molecules, exhibiting anti-proliferation effects on these cells. For example, exopolysaccharides produced by *L. casei* SB27 inhibited the proliferation of HT-29 cells, and ferrichrome in the supernatant of *L. casei* ATCC334 culture induced apoptosis in colon cancer cells [51,52]. This evidence suggests that there are also other bacterial components or metabolites involved in the anti-proliferative effects of *L. casei*. Moreover, it was reported that *L. Casei* Lcr35 prevented adjuvant 5-FU chemotherapy-induced intestinal mucositis in colorectal cancer-bearing mice [53]. With all these anti-proliferative effects mediated by many molecules from *L. casei*, this bacterium is of potential significance for the future treatment of cancer. The MucBP protein is also found in *Lactobacillus acidophilus, Lactobacillus fermentum*, and *Lactobacillus fermentum*; the role of this protein in these bacteria-host interaction should also be investigated in the future. In all, our study offers an insight of how probiotics interact with their hosts via other ways than microbial metabolites. 

## Data Availability

Data are contained within the article.
